# PTHrP‐Producing Renal Cell Carcinoma Presenting as Rapidly Progressive Cognitive Impairment: A Case Report

**DOI:** 10.1002/iju5.70142

**Published:** 2026-01-20

**Authors:** Fumiakira Yano, Norifumi Sawada, Norikazu Tanaka, Koshiro Hikawa, Keiichiro Hirose, Yuko Ohtake, Takahiko Mitsui

**Affiliations:** ^1^ Department of Urology University of Yamanashi Chuo Japan

**Keywords:** case report, cognitive impairment, paraneoplastic hypercalcemia, parathyroid hormone‐related peptide, renal cell carcinoma

## Abstract

**Introduction:**

Humoral hypercalcemia of malignancy (HCM) caused by parathyroid hormone–related peptide (PTHrP) is a common paraneoplastic syndrome, and renal cell carcinoma (RCC) is one of the main causes. However, initial presentation with rapidly progressive cognitive decline is rare.

**Case Presentation:**

A 68‐year‐old woman was presented with a rapidly progressive cognitive impairment. Laboratory tests revealed severe hypercalcemia with elevated PTHrP and suppressed parathyroid hormone levels. Thoracoabdominal computed tomography revealed an 8‐cm left renal mass, which was pathologically diagnosed as a sarcomatoid RCC. She underwent left radical nephrectomy, after which serum calcium normalized and cognitive function improved markedly (Hasegawa Dementia Scale‐Revised score 7→28).

**Conclusion:**

This case demonstrates that an unexplained cognitive decline may be the first sign of PTHrP‐producing RCC. Checking serum calcium levels is essential, and surgical removal of the tumor can reverse both metabolic and neurological symptoms.

## Keynote Message

This case highlights that renal cell carcinoma can initially present with neuropsychiatric symptoms due to PTHrP‐mediated hypercalcemia. Early recognition of paraneoplastic hypercalcemia is critical for timely diagnosis and treatment. Surgical removal of the tumor can normalize calcium levels and lead to rapid cognitive recovery.

## 1 Introduction

Hypercalcemia of malignancy (HCM) is a common paraneoplastic syndrome, most frequently caused by parathyroid hormone–related peptide (PTHrP), which accounts for 38%–69% of HCM cases [1]. Patients typically present with confusion, nausea, vomiting, and dehydration in the context of markedly elevated serum calcium levels [2]. However, presentation with progressive cognitive impairment as the initial symptom is rare.

Here, we describe a case of PTHrP‐producing renal cell carcinoma (RCC) in a patient who presented with rapidly progressive cognitive impairment, highlighting the importance of hypercalcemia screening for the differential diagnosis of cognitive decline.

## 2 Case Presentation

A 68‐year‐old woman, who had been healthy before, visited our neurology department with a one‐month history of worsening cognitive problems. Initially, her family noticed frequent mistakes when using her smartphone. Over time, she became disoriented and experienced memory lapses. She also lost her appetite, developed mild motor decline, exhibited delusions, and developed urinary incontinence. At a local clinic, her Hasegawa Dementia Scale‐Revised (HDS‐R) score was 7/30; therefore, she was referred to our hospital.

On admission, her consciousness level was classified as I‐2 on the Japan Coma Scale (JCS), and she had mild disorientation. Brain computed tomography (CT) and magnetic resonance imaging did not reveal a stroke or tumor, and only mild atrophy was observed in the right medial temporal lobe, which was not very specific. Blood tests (Table 1) showed marked hypercalcemia (17.6 mg/dL; normal 8.5–10.5 mg/dL). PTHrP was high (14.4 pmol/L; normal < 2.0), and PTH level was suppressed. These findings suggested humoral hypercalcemia of malignancy.

**TABLE 1:** Laboratory results before and after surgery.

Laboratory test	Admission day	Pre‐operative day	Post‐operative day 1	Post‐operative day 40
Ca (mg/dL)	17.6	10.5	9.1	9.8
PTHrP (pmol/L)	14.4	NA	< 1.1	NA
Albumin (g/dL)	2.4	1.9	2.3	4.6
Creatinine (mg/dL)	1.88	0.85	1.01	0.75
eGFR (mL/min/1.73m^2^)	21	51	54	59
CRP (mg/dL)	11.7	4.98	6.95	0.1
Hemoglobin (g/dL)	7.4	8.5	10.8	12.3
WBC (/μL)	8660	5150	6930	5490
Platelets (/μL)	310 000	297 000	193 000	194 000

**FIGURE 1:**
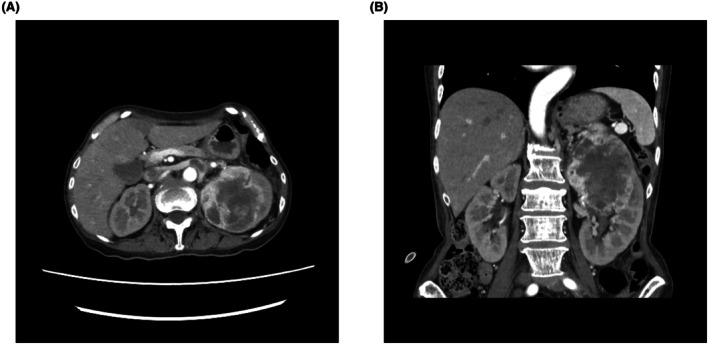
Abdominal computed tomography (CT) scans showing the left renal mass. (A) Axial view of a contrast‐enhanced CT scan showing a large, well‐defined mass (approximately 8 cm in diameter) in the left kidney. (B) Coronal view of the CT scan showing the left renal mass.

**FIGURE 2:**
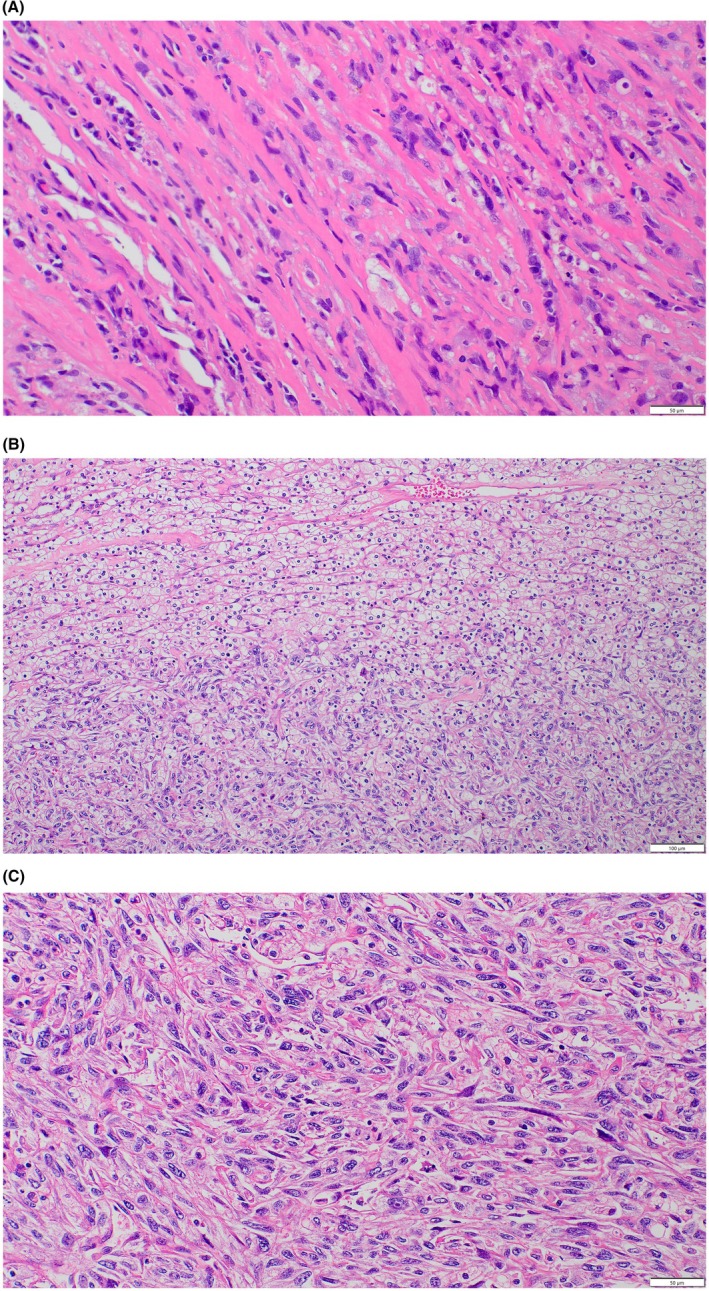
Histopathological findings of the resected tumor. (A) High‐power view showing pleomorphic, spindle‐shaped cells characteristic of sarcomatoid differentiation in a biopsy specimen. (H&E staining, original magnification ×400; scale bar = 50 μm). (B) Low‐power view of the resected specimen showing the coexistence of clear cell carcinoma and sarcomatoid features. (H&E staining, original magnification ×200; scale bar = 100 μm). (C) High‐power view of the resected specimen, showing a sarcomatoid component with pleomorphic spindle‐shaped cells. (H&E staining, original magnification ×400; scale bar = 50 μm).

We did contrast‐enhanced thoracoabdominal CT to search for a primary lesion. It revealed an 8‐cm mass in the left kidney with strong enhancement (Figure 1), suggestive of renal cell carcinoma. The patient also had severe anemia (Hb 7.4 g/dL) and low albumin (2.4 g/dL). Before surgery, intravenous fluids, zoledronic acid, and blood transfusions were administered. These supportive measures resulted in partial and temporary improvement in serum calcium levels, but definitive normalization was achieved only after tumor resection. After the patient's condition stabilized, we performed a CT‐guided biopsy, which confirmed sarcomatoid renal cell carcinoma (Figure 2A). No distant metastases were observed, and the clinical stage was cT3aN0M0. Just before surgery, the serum calcium level had decreased to 10.5 mg/dL.

**FIGURE 3:**
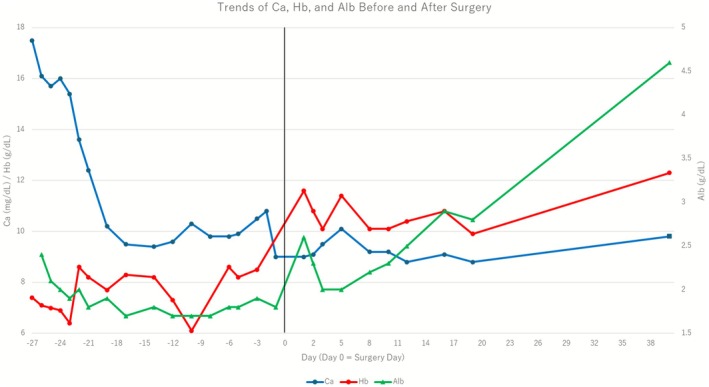
Trends of serum calcium, hemoglobin, and albumin before and after surgery. Serum calcium (Ca), hemoglobin (Hb), and albumin (Alb) before and after surgery. The *x*‐axis shows the number of days relative to surgery (day 0). The curves illustrate the normalization of calcium levels and the subsequent improvement in Hb and Alb levels after tumor removal.

Left radical nephrectomy was performed via an open approach, including resection of the renal vein with the tumor thrombus. On the first postoperative day, serum PTHrP levels decreased to below the detection limit (< 1.1 pmol/L, Table 1), and serum calcium levels were normal. Pathological examination revealed a clear cell carcinoma with sarcomatoid features (Figure 2B,C). Postoperatively, her cognitive function quickly improved. The HDS‐R score increased to 17 one week later, and memory loss and confusion almost disappeared. Three months after the nephrectomy, adjuvant pembrolizumab therapy was initiated. At 6 months follow‐up, her HDS‐R score was 28 and there was no recurrence. Hemoglobin and albumin also gradually improved (Figure 3, Table 1). She also regained sufficient physical and cognitive function to enjoy hiking again, indicating a good overall quality of life.

## 3 Discussion

Rapidly progressive cognitive impairment as the initial manifestation of PTHrP‐producing RCC has rarely been reported, and only a limited number of cases in the literature have described neuropsychiatric symptoms as the presenting feature of hypercalcemia. This case highlights two key lessons: first, screening for hypercalcemia in patients with unexplained cognitive impairment is important. Second, surgical resection of the primary tumor is crucial for resolving paraneoplastic syndrome.

In previous study, 28%–36% of hypercalcemia cases have been attributed to malignancy [3]. Among patients with known malignancy who develop hypercalcemia, the most common malignancies are lung and kidney [4]. The pathophysiology of HCM can be broadly classified into three mechanisms: (1) Local osteolytic hypercalcemia, (2) Humoral hypercalcemia mediated through PTHrP; and (3) excess production of 1,25‐dihydroxy vitamin D [5]. In this case, RCC with sarcomatoid changes was detected by biopsy, and elevated PTHrP and suppressed PTH were observed, indicating hypercalcemia caused by PTHrP‐producing renal cell carcinoma. Although immunohistochemical confirmation of PTHrP expression in tumor tissue would ideally strengthen the diagnosis, this analysis was not performed in the present case because no validated antibody was available at our institution at the time of diagnosis. However, the diagnosis of PTHrP‐producing renal cell carcinoma is strongly supported by the markedly elevated serum PTHrP level with suppressed intact parathyroid hormone, together with its rapid normalization immediately after nephrectomy. In addition, serial postoperative measurements of PTHrP were limited in this case, as additional samples were not obtained after postoperative day 1 once serum calcium levels had normalized. This represents a limitation of the present report. An important feature of the present case is the markedly elevated PTHrP level despite the absence of distant metastasis. Sarcomatoid differentiation in RCC represents highly aggressive tumor biology and is frequently associated with paraneoplastic manifestations, including cytokine or humoral factor production. Therefore, excessive PTHrP secretion can occur even in clinically localized disease. In addition, hypercalcemia itself is recognized as an adverse prognostic factor in major prognostic models for RCC, such as the MSKCC and IMDC classifications, further suggesting aggressive biological behavior in this case. Generally, hypercalcemia affects multiple organ systems, and the severity of symptoms varies. Milder symptoms of hypercalcemia include general malaise, constipation, nausea, and decreased ability to concentrate. Severe neurological manifestations, include muscle weakness, stupor, and coma [6]. This patient presented with rapidly progressing cognitive impairment, prompting consultation, making it a rare case. The critical branching point in the diagnostic workup of hypercalcemia is PTH measurement to determine whether hypercalcemia is PTH‐mediated. If hypercalcemia is not PTH‐mediated, then the evaluation includes the measurement of PTHrP, TSH, 25(OH) vitamin D, and 1,25(OH)2 vitamin D [6].

Patients with HCM are frequently malnourished and have poor performance status; therefore, one might conclude that they are poor candidates for surgery. However, previous reports have shown that surgical resection of the primary tumor in cases of HCM and primary hyperparathyroidism can markedly improve hypercalcemia, leading to recovery of both physical and mental function [7, 8]. This patient also showed severe anemia and hypoalbuminemia, but the surgery resulted in dramatic improvement. In patients with advanced or metastatic RCC, cytoreductive nephrectomy may be considered in selected patients with good performance status, while systemic therapy including immune checkpoint inhibitors plays a central role in controlling both tumor burden and paraneoplastic hypercalcemia. Bisphosphonates or denosumab can also be used as supportive measures to manage severe hypercalcemia.

## 4 Conclusion

In this case, PTHrP‐producing renal cell carcinoma presented with rapidly progressive cognitive decline. This highlights the importance of checking for hypercalcemia in patients with unexplained cognitive decline and supports early tumor control when PTHrP‐mediated disease is suspected.

## Ethics Statement

Not applicable. This case report did not require ethical approval under the policy of Yamanashi University Hospital.

## Consent

Written informed consent was obtained from the patient for publication of this case report and accompanying images.

## Conflicts of Interest

The authors declare no conflicts of interest.

## Data Availability

Data sharing not applicable to this article as no datasets were generated or analyzed during the current study.
